# Single cell transcriptomics of bone marrow derived macrophages reveals Ccl5 as a biomarker of direct IFNAR-independent responses to DNA sensing

**DOI:** 10.3389/fimmu.2023.1199730

**Published:** 2023-05-18

**Authors:** Emily McCarty, Justin Yu, Van K. Ninh, David M. Calcagno, Jodi Lee, Kevin R. King

**Affiliations:** ^1^ Department of Bioengineering, Jacobs School of Engineering, University of California San Diego, La Jolla, CA, United States; ^2^ Division of Cardiovascular Medicine, Department of Medicine, University of California San Diego, La Jolla, CA, United States

**Keywords:** dsDNA, Irf3, Ifnar, interferon stimulated genes, single cell RNA-Seq, transcriptomics, macrophages, Ccl5

## Abstract

**Introduction:**

The type I interferon (IFN) response is an innate immune program that mediates anti-viral, anti-cancer, auto-immune, auto-inflammatory, and sterile injury responses. Bone marrow derived macrophages (BMDMs) are commonly used to model macrophage type I IFN responses, but the use of bulk measurement techniques obscures underlying cellular heterogeneity. This is particularly important for the IFN response to immune stimulatory double-stranded DNA (dsDNA) because it elicits overlapping direct and indirect responses, the latter of which depend on type I IFN cytokines signaling *via* the IFN alpha receptor (IFNAR) to upregulate expression of interferon stimulated genes (ISGs). Single cell transcriptomics has emerged as a powerful tool for revealing functional variability within cell populations.

**Methods:**

Here, we use single cell RNA-Seq to examine BMDM heterogeneity at steady state and after immune-stimulatory DNA stimulation, with or without IFNAR-dependent amplification.

**Results:**

We find that many macrophages express ISGs after DNA stimulation. We also find that a subset of macrophages express ISGs even if IFNAR is inhibited, suggesting that they are direct responders. Analysis of this subset reveals *Ccl5* to be an IFNAR-independent marker gene of direct DNA sensing cells.

**Discussion:**

Our studies provide a method for studying direct responders to IFN-inducing stimuli and demonstrate the importance of characterizing BMDM models of innate immune responses with single cell resolution.

## Introduction

The type I interferon (IFN) response is an innate immune program that fuels inflammation during diverse pathologic processes including infections, malignancies, sterile tissue injury, autoimmunity, and autoinflammatory diseases (1–9). In the setting of viral infections, this response is adaptive; however, in the context of sterile injuries and autoimmune diseases, this response is often overly exuberant and maladaptive. IFNs are thought to mediate pathology by signaling *via* their cell surface IFN alpha receptor (IFNAR) as detailed below. As a result, therapies based on inhibition of type I IFN signaling using neutralizing antibodies against the IFN alpha receptor (INFAR) have been developed and were recently FDA approved for treatment of lupus (10).

**Figure 1:**
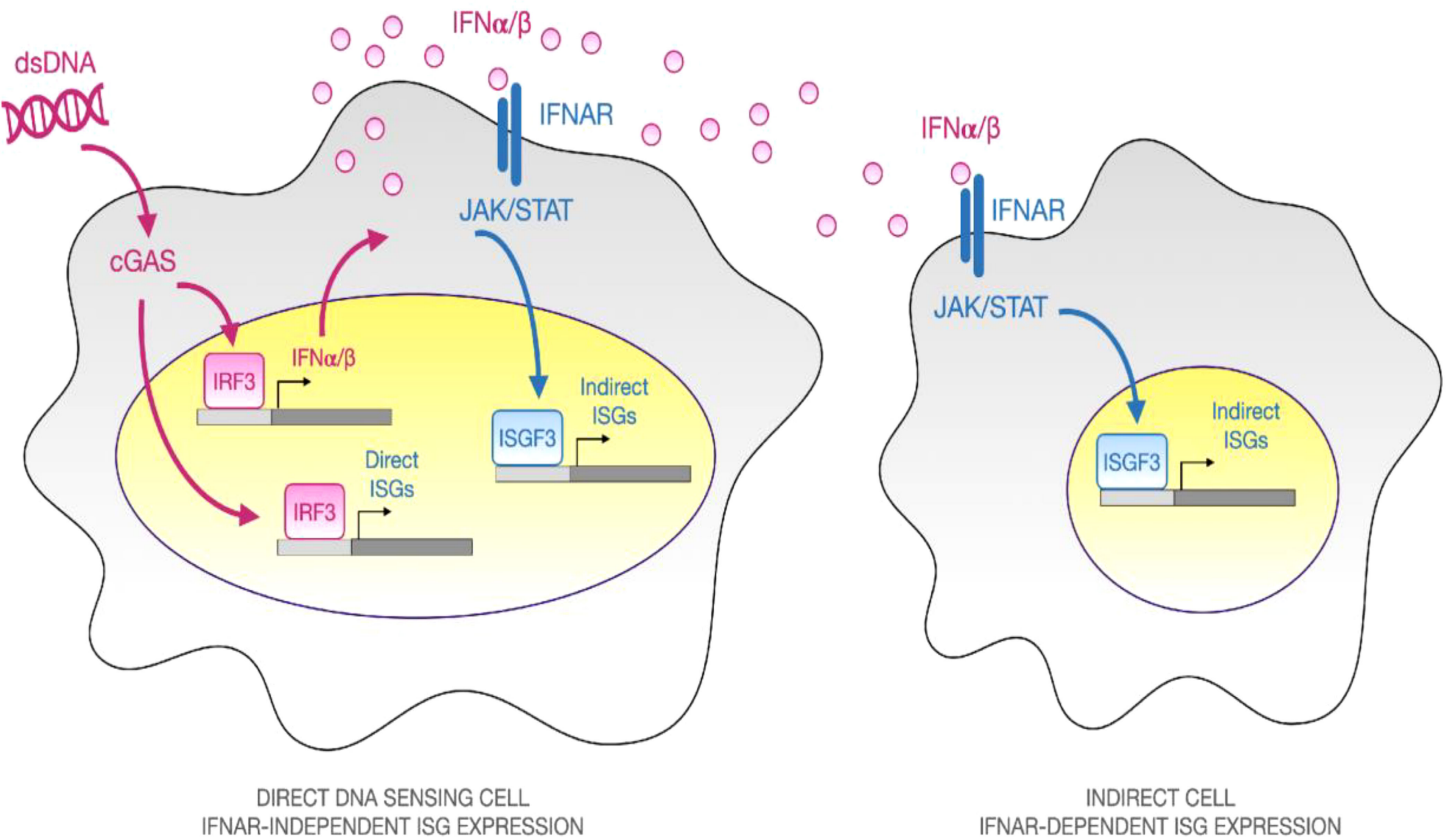
Overview of Direct and Secondary Type I IFN response to Immune-stimulatory dsDNA. When double-stranded DNA (dsDNA) is complexed with a polyelectrolyte transfection reagent and added to cells in culture, it activates an innate immune response. Some cells internalize the particulate material, allowing dsDNA to be sensed by the cytosolic pattern recognition receptor cGAS, which catalyzes synthesis of the second messenger, cGAMP that signals *via* the adaptor STING and the TBK1 kinase to promote phosphorylation, dimerization, and nuclear translocation of the master transcriptional regulator IRF3. Once inside the nucleus, IRF3 binds to the promoters of IRF3-dependent genes such as the type I interferon cytokines, IFNα and IFNβ. Secreted type I IFN cytokines can diffuse to neighboring cells and bind to their cognate surface receptor, interferon alpha receptor (IFNAR), leading to JAK/STAT signaling and assembly of a heterotrimeric transcriptional regulatory complex known as ISGF3, composed of Stat1, Stat2, and Irf9. ISGF3 binds to ISRE consensus sequences and upregulates hundreds of effector genes, which are collectively known as interferon stimulated genes (ISGs). By performing experiments in the presence or absence of an IFNAR neutralizing antibody, one can dissect the “Direct” IFNAR-independent and “Indirect” IFNAR-dependent elements of the type I IFN response.

The type I IFN response is shown schematically in **Figure 1**. It begins when molecularly conserved pathogen- and damage- associated molecular patterns (DAMPs and PAMPs) are detected by genome encoded pattern recognition receptors (PRRs), for example, when immune stimulatory double-stranded DNA (dsDNA) is sensed by the cytosolic DNA sensor intracellular cyclic GMP-AMP synthase (cGAS) or Toll Like Receptor 9 (TLR9) (7, 11, 12). Receptor engagement leads to intracellular signaling that culminates in activation of the master transcriptional regulator interferon response factor 3 (IRF3) which, once phosphorylated, dimerizes, and translocates into the nucleus where it induces expression of target genes such as type I IFN secreted cytokines (Ifnα/β) (7, 11, 12). Beyond the cGAS, other cytosolic DNA sensors have been described but they are not known to induce type I IFN responses (13–15).

Type I IFNs are diffusible extracellular cytokines that spread responses from directly-stimulated cells to neighboring cells by binding to cell surface interferon alpha receptors (IFNAR1 and IFNAR2, hereafter IFNAR) (**Figure 1**). Autocrine signaling is also possible if the directly-stimulated cells express IFNAR. Ligand binding of IFNAR leads to intracellular signaling that activates a heterotrimeric transcriptional regulatory complex composed of STAT1, STAT2, and IRF9, called ISGF3. Within the nucleus, ISGF3 upregulates hundreds of genes that are collectively referred to as interferon stimulated genes (ISGs). Some ISGs are thought to be directly inducible by IRF3, independent of IFNAR, while others are IFNAR-dependent, but it is challenging to separate and quantify direct and indirect effects using bulk measurement techniques (16–18). Most experimental studies of the type I IFN response to immune stimulatory DNA rely on bulk measurement techniques that average responses over large numbers of cells. It is therefore difficult to separate, characterize, and quantify the responses of direct DNA-sensing cells amidst secondary IFNAR-dependent cells. Even when IFNAR Abs are used to block secondary signaling, the primary response is averaged across all cells and is thus severely diluted.

Here, we utilize single cell RNA-Seq to characterize bone marrow derived macrophages (BMDMs), a commonly used *in vitro* model of tissue macrophages, at steady state and after exposure to immune stimulatory dsDNA with or without simultaneous IFNAR Ab treatment or genetic deficiency of IFNAR (19). This enables dissection of IFNAR-dependent and IFNAR-independent responses with single cell resolution, even when cells derive from the same culture well and thus share a microenvironment. Our results point to *Ccl5*, a proinflammatory chemokine, as a marker of direct DNA sensing cells which is inducible independent of IFNAR-mediated amplification.

## Results

### Single cell analysis of bone marrow derived macrophage subsets

**Figure 2:**
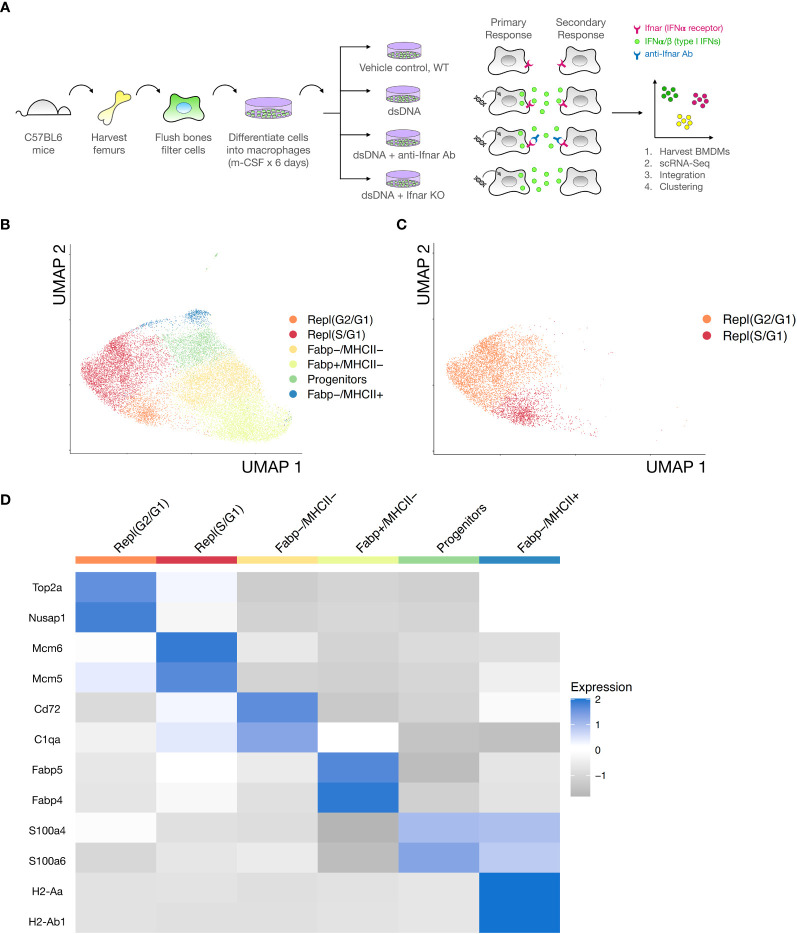
Integrated single cell transcriptomes reveal cell-cycling and non-cycling BMDM subsets. **(A)** Bone marrow derived macrophages (BMDMs) were isolated from adult male C57BL6 and IFNAR KO mice and differentiated in culture with m-CSF. BMDM were stimulated for 24 hours with one of the following i) vehicle control, ii) dsDNA complexed to LTX transfection reagent, or iii) dsDNA + IFNAR Ab, or iv) dsDNA-transfected IFNAR KO. Cells were then collected, stained with DAPI, FACS sorted to isolate live single cells, and processed for single cell RNA-Seq barcoding and sequencing using established 10X Genomics protocols. Condition 1 was designed to define the transcriptional heterogeneity and BMDM subsets in the unstimulated state. Condition 2 was designed to define the transcriptional response of BMDM subsets to dsDNA, an inducer of primary and secondary type I IFN responses. Condition 3 was designed to block IFN-dependent secondary responses and isolate the direct effects of dsDNA. **(B)** Data from all three experimental conditions, excluding the IFNAR KO due to strain differences, was integrated and clustered. Unsupervised clustering of integrated data (n= 19,343cells) revealed at least 6 distinct BMDM subsets. Data is dimensionally reduced using UMAP and displayed on a 2D plot to communicate relative similarities in transcriptional profiles between BMDM subsets. **(C)** Identification of replicating BMDMs from clusters based on cell cycle phase (color-coded legend) using cell cycle sorting. **(D)** Heatmap of biological replicates averaged, scaled expression defining differentially expressed genes for each BMDM subset. Cell cycling subsets (clusters 1-2, red & orange) and non-cycling subsets (clusters 3-6, yellow, green, and blue) are annotated.

To dissect direct and indirect responses to transfected immunogenic DNA stimulation with single cell resolution, we designed the following experiment. We used single cell RNA-Seq to define the heterogeneity of cultured BMDMs (i) at steady state, (ii) in response to dsDNA alone, or (iii) in response to dsDNA + IFNAR Ab inhibition of secondary signaling, or (iv) in dsDNA-treated IFNAR KO (**Figure 2A**). BMDMs were derived by flushing hematopoietic cells from the femurs of adult C57BL6 mice and differentiating them into macrophages in culture using monocyte colony stimulating factor (m-CSF) as we previously described (20). We then subjected the cultures to 24 hours of stimulation with the conditions above, after which BMDMs were collected, stained with DAPI, purified by flow cytometry, and subjected to droplet-based microfluidic single cell barcoding (Chromium, 10X Genomics), library preparation, and next generation sequencing (NovaSeq, Illumina). After demultiplexing and mapping to a reference murine genome, we performed shared nearest neighbor (SNN) clustering on the integrated data from all three treatment groups to define a universal embedding of shared BMDM subtypes (**Figure 2B**). Unbiased clustering revealed 6 coarse BMDM clusters, 2 of which were interpreted to be proliferating based on expression of canonical cell replication marker genes and labeled them Repl(G2/G1) and Repl(S/G1) (**Supplementary Figure S1**). Proliferating BMDMs were stratified by cell cycle phase cell based on their expression of *Mcm6* and *Mcm5* (S phase), *Top2a* and *Cenpa* (G1 phase), *Birc5* and *Nusap1* (G2 phase) (**Figures 2C, D**). Non-proliferating BMDMs were also readily stratified into 3 clusters based on their expression of *Fabp4* and *Fabp5* and MHCII genes such as *H2-Aa* and *H2-Ab1* (**Figures 2C, D**). We labeled the non-proliferating BMDM subsets (*Fabp4-,MHCII-)*, (*Fabp4+, MHCII-)*, and (*Fabp4+, MHCII+).* The final cluster was defined as a monocyte-like BMDM Progenitor characterized by expression of *S100a4* and *S100a6*.

### Single cell analysis of cytosolic DNA-induced ISG expression in BMDMs

**Figure 3:**
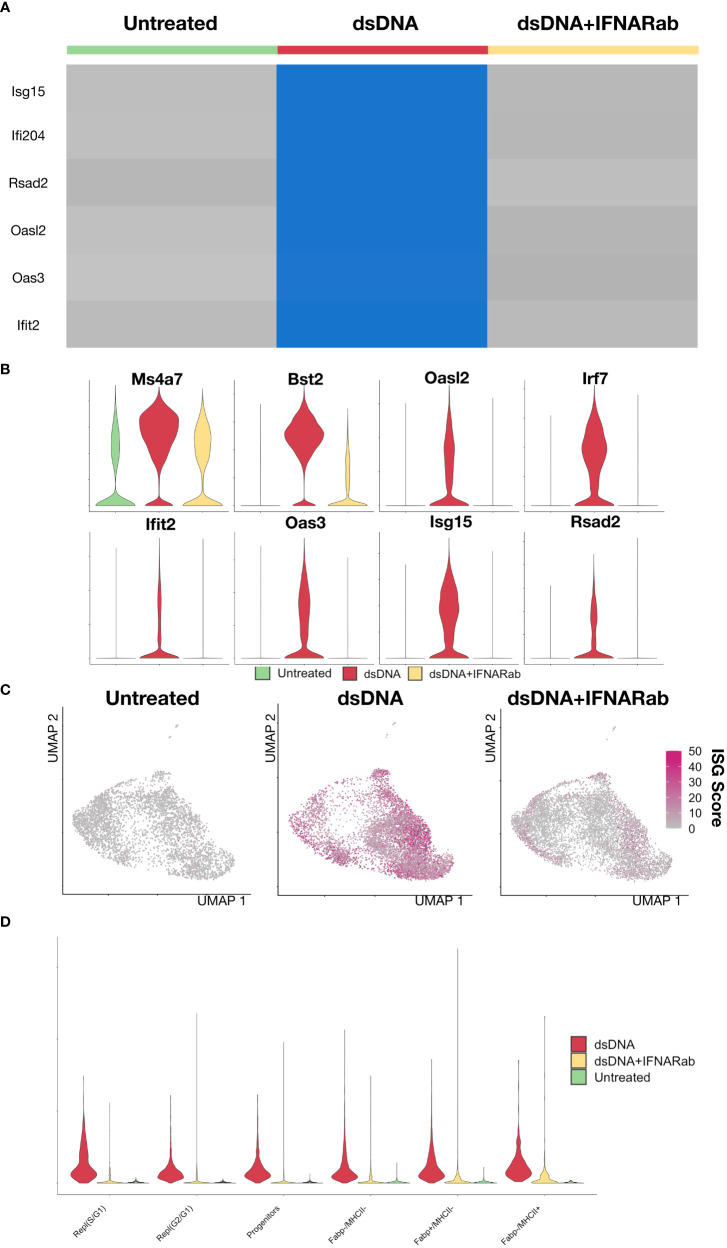
Immune stimulatory DNA induces a type I IFN response that can be partially blocked by IFNAR Ab **(A)** Heatmap of of biological replicates averaged, scaled expression of ISG genes sorted by experimental condition (1: control, 2: dsDNA, 3: dsDNA+IFNAR Ab). **(B)** Violin plot of *Ms4a7*, a macrophage marker gene, and several ISGs (*Bst2, Oasl2, Irf7, Ifit2, Oas3, Isg15, Rsad2*). **(C)** An ISG Score defined as the summed expression of canonical ISGs was calculated for each cell and displayed as magenta intensity on a UMAP feature plot for each condition. **(D)** A violin plot of ISG Score was plotted across BMDM subsets and split by experimental condition.

Having defined the BMDM subtypes at steady state, we turned our attention to the response to dsDNA. Experiments based on bulk measurement techniques (e.g., qPCR) have firmly established that dsDNA complexed with a transfection reagent, when delivered to macrophages in culture, induces expression of type I IFNs and ISGs (21). However, underlying single cell heterogeneity remains incompletely defined. Consistent with results from bulk experiments, single cell RNA-Seq profiling of dsDNA-stimulated BMDMs markedly induced expression of ISGs (Irf7, *Ifit2, Oasl2, Rsad2, Isg15*) compared to unstimulated controls as well as compared to dsDNA in the context of IFNAR Ab, thus demonstrating successful inhibition of secondary amplification (**Figure 3A**). Violin plots confirmed dsDNA-dependent and IFNAR-dependent induction of ISGs at the single gene level while broader macrophage marker genes such as Ms4a7 remained highly expressed across all three conditions (**Figure 3B**). To evaluate dsDNA responses independent of clustering, we created an ISG Score based on the summed expression of a collection of ISGs derived from previously published studies (**Table 1**). dsDNA-treated BMDMs had the highest ISG Scores, which were almost completely abrogated by IFNAR Ab inhibition (**Figure 3C**). Interestingly, we did not find that dsDNA induced ISGs preferentially expressed by one or more of the BMDM subtypes. Instead, they were uniformly elevated across all proliferative and non-proliferative subsets compared to control (**Figure 3D**). Although we included IFNAR KO BMDMs, they were found to have significant ISG expression in scattered cells with or without DNA stimulation, which limited their utility for identifying direct DNA-stimulated cells (**Supplementary Figure S2**). Taken together, these data demonstrated that dsDNA stimulation induces ISG expression in both proliferating and non-proliferating BMDMs, and that secondary amplification of the response *via* type I IFN secretion plays a dominant role in the ISG response since it is markedly abrogated by simultaneous blockade with a neutralizing IFNAR Ab.

**Table 1:** ISGs used in ISG score.

ISG		
***Ifi204***		
***Isg15***		
***Irf7***		
***Ifit1***		
***Rsad2***		
***Oasl2***		
***Oas3***		
***Gpb5***		
***Bst2***		
***Ifit2***		
***Ifit3***		
***Oas2***		
***Isg20***		
***Cxcl10***		
***Oasl1***		
***Gpb2***		
***Ccl5***		
***Ccl3***		

### Direct IFNAR-independent responses to immune stimulatory DNA

**Figure 4:**
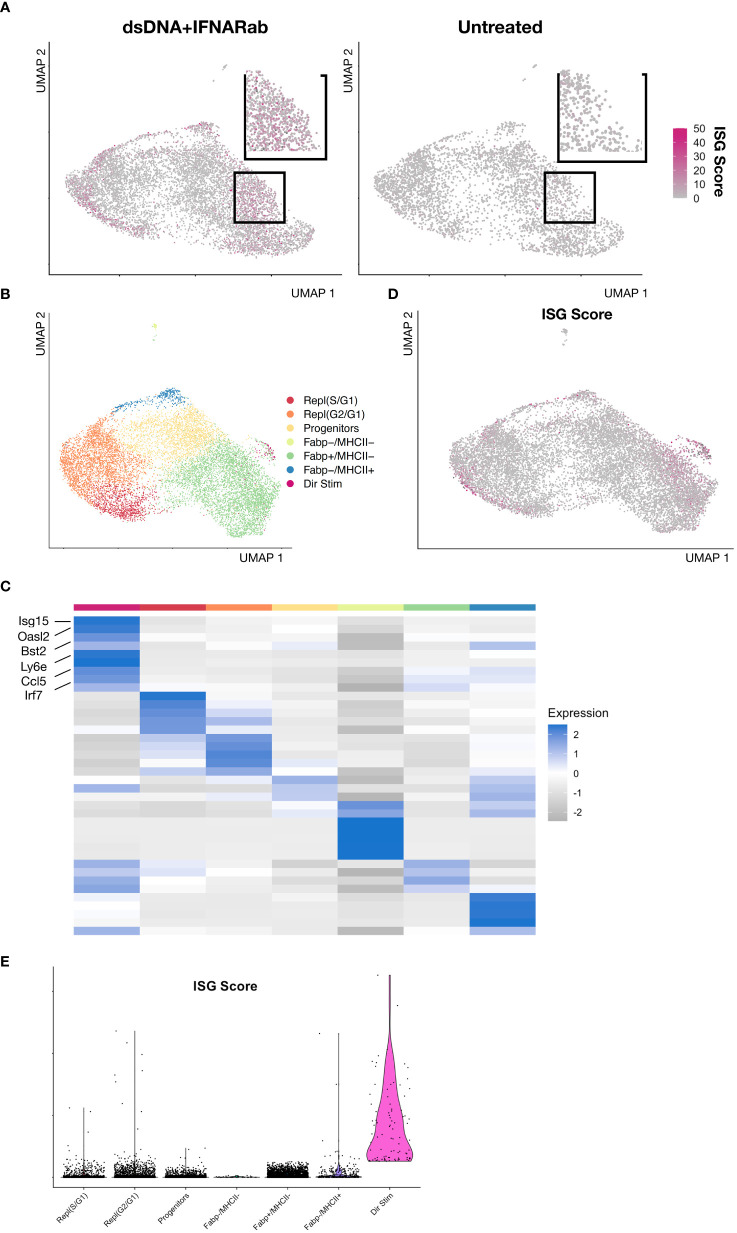
IFNAR-independent BMDM responses to immune stimulatory DNA **(A)** Feature plot of dsDNA+IFNAR Ab and untreated experimental conditions colored magenta based on ISG Score. **(B)** Feature plot of integrated and clustered data from dsDNA+IFNAR Ab and Untreated conditions, demonstrating BMDM subsets. **(C)** Heatmap of biological replicates averaged, scaled expression displaying marker genes for each subset. **(D)** Feature plot of ISG Score reveals a small cluster expressing ISGs amidst a larger cluster of Fabp4+MHCII- cluster. **(E)** Violin plot of ISG Score for each BMDM subset from the integration of control and dsDNA+IFNAR Ab conditions.

Since IFNAR Ab treatment markedly inhibited secondary amplification of DNA-induced IFN responses, we reasoned that any residual ISG expression must result from IFNAR-independent responses to DNA. To identify direct DNA-sensing cells, we bioinformatically combined BMDMs from control and IFNAR Ab treated cells and reclustered (**Figure 4A**, **Supplementary Figure S3**). This revealed a small group of BMDMs with high ISG scores despite inhibition of IFNAR-dependent amplification (**Figure 4B**). We interpreted these ISG-expressing BMDMs to be direct responders to dsDNA stimulation. Unbiased clustering of IFNAR Ab-treated Condition 3 cells revealed 8 distinct clusters including one expressing ISGs embedded within the broader Fabp4+MHCII- cluster, as illustrated in a feature plot (**Figure 4C**) and heatmap of top marker genes expressed by each cluster (**Figure 4C**). The top marker genes for directly DNA-stimulated BMDMs were *Ccl5, Irf7, Ifit1, Isg15, Rsad2, Oasl2, Bst2*, and *Ifi204* (**Figure 4D**). To confirm the result independent of clustering, we plotted the ISG Score for each DNA- and IFNAR-Ab-treated BMDM of Condition 3 and identified the same small population of Fabp4+MHCII-ISG+ cells (**Figure 4E**). Together, these results suggest that direct DNA-sensing BMDMs are preferentially Fabp4+MHCII- macrophages.

### Gene expression signatures of direct Ifnar-independent DNA-induced macrophages

**Figure 5:**
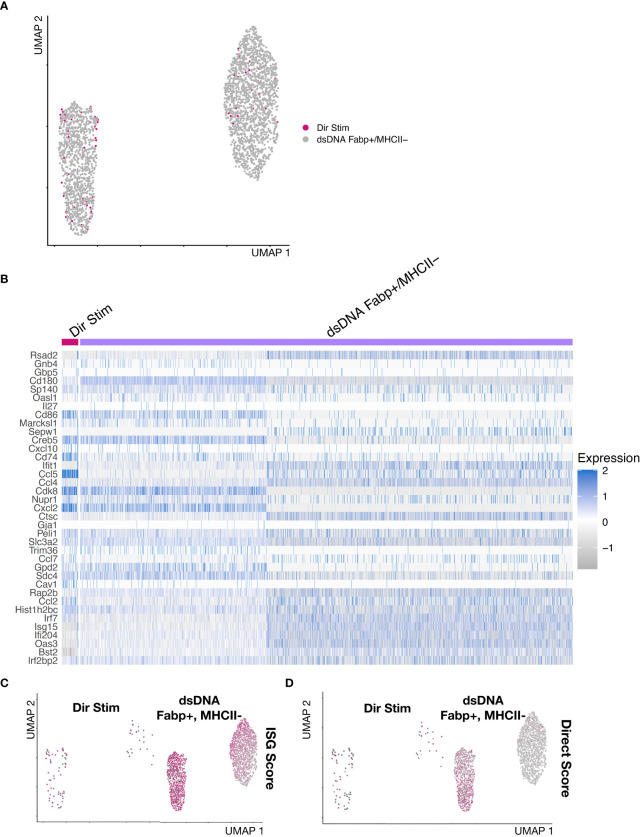
Gene expression signature of direct dsDNA-sensing IFNAR-independent BMDMs. **(A)** Reclustering of Direct Stimulated cells from dsDNA + IFNAR Ab condition integrated with Fabp4+MHCII- BMDMs subset from the dsDNA stimulated experimental condition. **(B)** Heatmap of Fabp4+MHCII- BMDMs reveals ISG+ and ISG- subsets. The ISG+ subset are the putative direct dsDNA-stimulated macrophages. Differentially expressed genes between ISG+ and ISG- subsets include *Ccl5,Cdk8, Cxcl2, and Cd74*. **(C, D)** Feature plots comparing **(C)** ISG Score compared to a **(D)** DirStim Score (*Ccl5, Cdk8, Cxcl2, and Cd74*).

Bulk measurement techniques do not allow separation of direct DNA-sensing cells from the more abundant secondary IFNAR-dependent cells. This limits separation of cell-specific roles in initiation and amplification of IFN responses *in vivo*. To identify genes that may discriminate direct and indirect cells, we identified the most highly differentially expressed genes between direct DNA responsive cells and secondary IFNAR-dependent cells. First, we isolated Fabp4+MHCII- macrophages, which contained the majority of directly responding cells. We reclustered them, which revealed an ISG+ and ISG- population. Next, we combined the direct macrophages from condition 3 (dsDNA + IFNAR Ab) with the Fabp4+MHCII- macrophages from condition 2 (dsDNA Alone) and reclustered (**Figure 5A**, **Supplementary Figure S4**). We displayed expression of 45 ISGs as a heatmap. This revealed *Ccl5* to be the most differentially expressed between the direct cells from condition 3 and combined indirect and direct cells from condition 2 (**Figure 5B**). Feature plots show the cells colored by ISG Score compared to the more stringent by Direct DNA-sensing Score (**Figure 5C**). We selected the top 4 genes and created a Direct Score (Ccl5, Cdk8, Cxcl2, and Cd74) (**Figure 5D**). Based on these results, we propose that the top gene, *Ccl5*, may represent a useful marker gene for identifying direct DNA sensing cells.

## Discussion

BMDMs are a widely used experimental tool used across diverse innate immune studies, but they are typically analyzed using bulk measurement techniques that obscure population heterogeneity (19). In this study we applied single cell RNA-seq to reveal functional heterogeneity within cultured BMDMs. Using single cell RNA-Seq, we show that immune stimulatory DNA induces ISG expression across all proliferating and non-proliferating BMDM subsets; however, in the context of anti-IFNAR Ab blockade, DNA was only able to induce a small non-proliferative Fabp4+MHCII- population of macrophages. These cells, which do not benefit from secondary signaling *via* IFNAR, were interpreted as directly-stimulated cells. Using this population, we identified *Ccl5*, also known as RANTES, as a transcriptional biomarker that discriminates directly from indirectly DNA-stimulated cells. Ccl5 is a secreted proinflammatory chemokine capable of recruiting diverse immune cell types (17, 22, 23). However, independent of its functional role, we propose that *Ccl5* may be useful transcriptional biomarker for identifying direct DNA-stimulated cells *in vivo* using single cell RNA-Seq.

BMDMs are a ubiquitous part of an innate immunologist’s experimental toolkit, owing to the ease of harvesting and differentiating them in cell culture. However, an often-overlooked assumption of this model is that all cells in the culture are assumed to represent the response of a prototypical macrophage (19). Single cell transcriptomics has emerged as a powerful tool for revealing cellular heterogeneity *in vitro* and *in vivo* within population previously assumed to be homogeneous. *In vivo*, this technology is informing the growing appreciation of macrophage diversity within the bone marrow and at sites of tissue injury (3, 24). Here, we apply single cell transcriptomics to the commonly used BMDM model experimental system. At baseline, transcriptional profiling of BMDMs divides cultured macrophages into proliferative and non-proliferative subsets, each with additional substructure. Our data suggests that when BMDMs are stimulated with immune stimulatory DNA, it is the non-proliferating BMDMs that are directly stimulated, which then broadly recruit both proliferative and non-proliferative cells *via* secreted type I IFN cytokines signaling *via* the IFNAR cell surface receptor to express secondary ISGs (25, 26).

Limitations of our study include the small biological sample size; however, this is somewhat mitigated by the large number of total single cell transcriptomes analyzed (20,000+). An alternative explanation for the directly stimulated cells that we observed is that there is a population of cells that are resistant to IFNAR Ab blockade. While this cannot be disproven, it seems unlikely since the resistant population would have to also have to uniquely respond to DNA stimulation with overexpression of *Ccl5* compared to other ISGs when compared to all IFN-induced cells.

## Conclusion

In summary, we have used single cell RNA-Seq to define the heterogeneity of macrophages in culture, both at steady state and in response to immune stimulatory DNA, with and without blockade of secondary IFNAR-dependent amplification, revealing *Ccl5* as an IFNAR-independent marker of direct DNA-sensing cells.

## Methods

### Animals and tissue processing

Mouse experiments were approved and conducted under the oversight of University of California San Diego Institutional Animal Care and Use Committee (IACUC #17144) using Adult C57BL/6J (3 *WT mice*, stock 000664, and 2 IFNAR -/- mice, stock 028288). All experiments were performed with 10 to 14-week-old animals and were carried out using age and gender matched groups without randomization. All mice were maintained in a pathogen-free environment of UC San Diego.

### Cell culture

Bone marrow derived macrophages (BMDMs) were isolated by flushing femurs of adult mice and culturing the resulting cells in 10% FBS 1% Pen/Strep-containing DMEM supplemented with 10ng/mL recombinant m-CSF (Peprotech) for 7 days. 5μg of immunogenic HT DNA (*In vivo*gen) was complexed with a Lipofectamine transfection agent (ThermoFisher) at a ratio of 1:1.5 in serum free-media and added to 1 million BMDMs in a 6-well multi-well plate with serum- and mCSF-containing media for each experiment. Two independent experiments were performed, yielding n = 5 for each condition. For inhibition of secondary signaling *via* the IFNAR receptor, cells were treated with 20μg/ml of MAR1-5A3 IFNAR neutralizing antibody or isotype control (BioXCell).

### Flow cytometry

Isolated cells were stained at 4°C in FACS buffer (PBS supplemented with 2.5% bovine serum albumin). Cell suspensions were labeled with DAPI just prior to flow cytometric analysis to allow exclusion of dead cells. Doublets and dead cells were excluded by forward scatter and Dapi. Data was acquired by Sony sorter MA900 at UCSD and analyzed with FlowJo software.

### Single cell RNA-seq

Single cell RNA-Seq was performed by microfluidic droplet-based encapsulation, barcoding, and library preparation (10X Genomics) as previously described (27). Paired end sequencing was performed on an Illumina NovaSeq instrument. Low level analysis, including demultiplexing, mapping to a reference transcriptome (Ensembl Release 85 - GRCm38.p5), and eliminating redundant UMIs, was performed with the CellRanger pipeline.

### Single-cell RNA-seq data quality control, normalization and integration

To account for variations in sequencing depth, total transcript count for each cell was scaled to 10,000 molecules, and raw counts for each gene were normalized to the total transcript count associated with that cell and then natural log transformed. Cells with between 200 and 2,000 uniquely expressed genes and < 1% mitochondrial counts were retained for further analysis. Highly variable genes across individual datasets were identified with the *FindVariableFeatures* method from the Seurat R package (version 3.0) by selecting 3,000 genes with the highest feature variance after variance-stabilizing transformation. Integration of multiple single-cell RNA-seq datasets was performed in Seurat to enable harmonized clustering and downstream comparative analyses across conditions (28–30). Anchoring cell pairs between datasets were identified by Canonical Correlation Analysis (CCA) and the mutual nearest neighbors (MNN) method using the Seurat *FindIntegrationAnchors* function.

### Dimensional reduction, unsupervised clustering, sub-clustering

After scaling and centering expression values for each variable gene, linear dimensionality reduction was performed on integrated data using principal component analysis (PCA). Clustering was performed using the shared nearest neighbor (SNN) clustering algorithm with the Louvain method for modularity optimization, as implemented in the Seurat *FindNeighbors* and *FindClusters* functions. To visualize data in two-dimensional space, Uniform Manifold Approximation and Projection (UMAP) dimensional reduction was performed. Differentially expressed genes (DEGs) between clusters were determined using a Wilcoxon Rank Sum test. Where specified, subsets of cells were isolated and reclustered to identify new DEGs.

### Quantification of ISG score and direct score

ISG Scores were measured as the sum of the raw reads for the ISGS: Ifi204, Isg15, Irf7, Ifit1, Rsad2, Oasl2, Oas3, Gpb5, Bst2, Ifit2, Ifit3, Oas2, Isg20, Cxcl10, Oasl1, Gpb2, Ccl5, Ccl3. Direct Scores were measured as the sum of the raw reads for: Ccl5, Cdk8, Cxcl2, and Cd74. ISG and Direct Scores were normalized to reads per cell and scaled by 10^4^.

### Statistics

Statistical analysis was performed using GraphPad Prism software. All data are represented as mean values +/- standard error of mean (S.E.M.) unless indicated otherwise. A statistical method was not used to predetermine sample size. All analyses were unpaired. *P* values are indicated by *P* values less than 0.05 were considered significant and are indicated by asterisks as follows: *p<0.05, **p<0.01, ***p<0.001, ****p<0.0001.

## Data availability statement

The single cell RNA-Seq datasets presented in this study have been deposited to the Gene Expression Omnibus under accession no. GSE229311 (GEO). The code used to process this data is publicly available at Zenodo (https://zenodo.org/record/7874451).

## Ethics statement

The animal study was reviewed and approved by University of California San Diego Institutional Animal Care and Use Committee (IACUC #17144).

## Author contributions

EM, JY, VN, and KK designed the study, performed analysis, and wrote the initial manuscript. VN conducted experiments. EM, JY, and DC performed analysis. KK directed the project. All authors contributed to the article and approved the submitted version.

## Supplementary material

Supplementary Figure 1 Integrated single cell transcriptomes reveal cell-cycling and non-cycling BMDM subsets. **(A)** Data from all experimental conditions was integrated and clustered. Unsupervised clustering of integrated data (n= 24,377 cells) revealed at least 6 distinct BMDM subsets. Data is dimensionally reduced using UMAP and displayed on a 2D plot to communicate relative similarities in transcriptional profiles between BMDM subsets. **(B)** Identification of replicating BMDMs from clusters based on cell cycle phase (color-coded legend) using cell cycle sorting. **(C)** Heatmap of biological replicates averaged, scaled expression of defining 1065 differentially expressed genes for each BMDM subset. Cell cycling subsets (clusters 1-2, red & orange) and non-cycling subsets (clusters 3-6, yellow, green, and blue) are annotated.

Supplementary Figure 2 Immune stimulatory DNA induces a type I IFN response that can be partially blocked by IFNAR Ab. **(A)** Heatmap of of biological replicates averaged, scaled expression of ISG genes sorted by experimental condition (1: control, 2: IFNAR KO control, 3: dsDNA, 4: dsDNA+IFNAR Ab, 5: dsDNA+IFNAR KO). **(B)** Violin plot of Ms4a7, a macrophage marker gene, and several ISGs (Bst2, Oasl2, Irf7, Ifit2, Oas3, Isg15, Rsad2). **(C)** An ISG Score defined as the summed expression of canonical ISGs was calculated for each cell and displayed as magenta intensity on a UMAP feature plot for each condition. **(D)** A violin plot of ISG Score was plotted across BMDM subsets and split by experimental condition.

Supplementary Figure 3 IFNAR-independent BMDM responses to immune stimulatory DNA. **(A)** Feature plot of dsDNA+IFNAR Ab, dsDNA+IFNAR KO, and untreated experimental conditions colored magenta based on ISG Score. **(B)** Feature plot of integrated and clustered data from dsDNA+IFNAR Ab, dsDNA+IFNAR KO, and Untreated conditions, demonstrating BMDM subsets. **(C)** Heatmap of biological replicates averaged, scaled expression displaying marker genes for each subset. **(D)** Feature plot of ISG Score reveals a small cluster expressing ISGs amidst a larger cluster of Fabp4+MHCII- cluster.**(E)** Violin plot of ISG Score for each BMDM subset from the integration of control and dsDNA+IFNAR Ab conditions.

Supplementary Figure 4 Gene expression signature of direct dsDNA-sensing IFNAR-independent BMDMs. **(A)** Reclustering of Direct Stimulated cells from dsDNA + IFNAR Ab and dsDNA+IFNAR KO conditions integrated with Fabp4+MHCII- BMDMs subset from the dsDNA stimulated experimental condition. **(B)** Heatmap of Fabp4+MHCII- BMDMs reveals ISG+ and ISG- subsets. The ISG+ subset are the putative direct dsDNA-stimulated macrophages. Differentially expressed genes between ISG+ and ISG- subsets include Ccl5, Cdk8, Cxcl2, and Cd74. C-D) Feature plots comparing **(C)** ISG Score compared to a **(D)** DirStim Score (Ccl5, Cdk8, Cxcl2, and Cd74).
