# Supplementary figures and images for: Single cell transcriptomics of bone marrow derived macrophages reveals Ccl5 as a biomarker of direct IFNAR-independent responses to DNA sensing
Source: Front Immunol. 2023 May 18;14:1199730. doi: 10.3389/fimmu.2023.1199730 (PMC10232813; doi:10.3389/fimmu.2023.1199730)

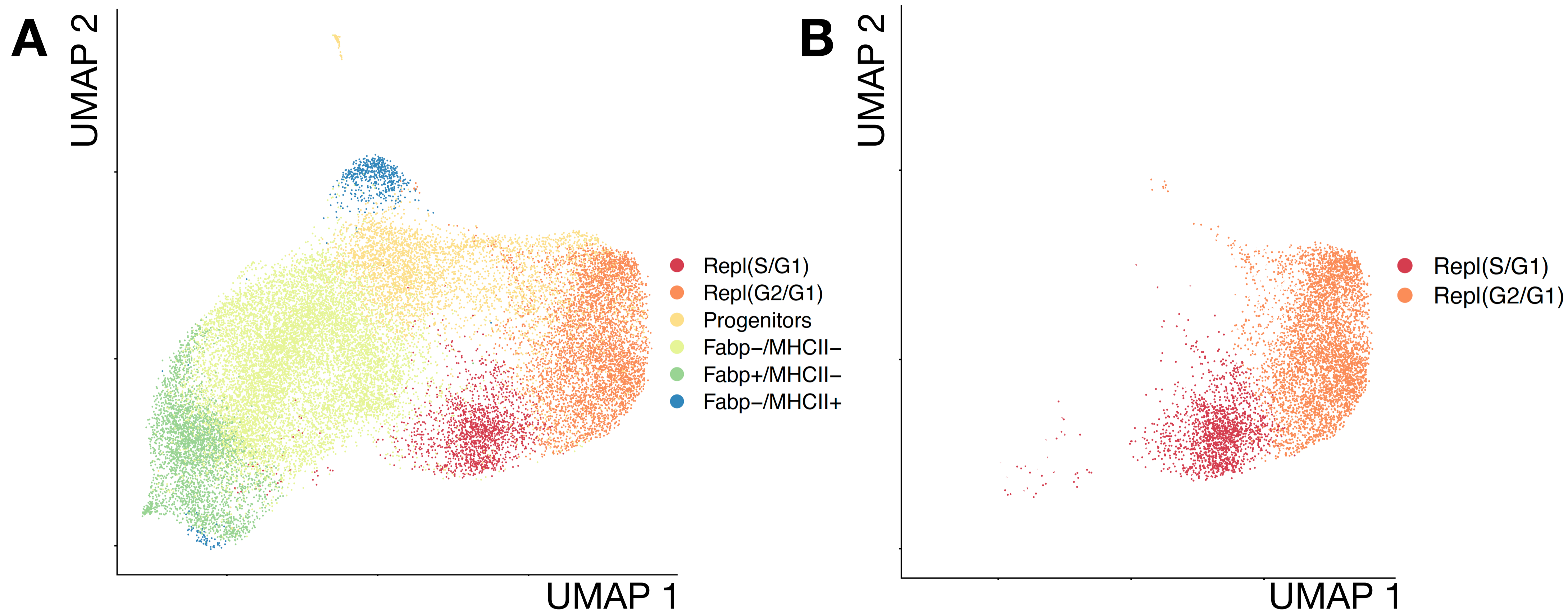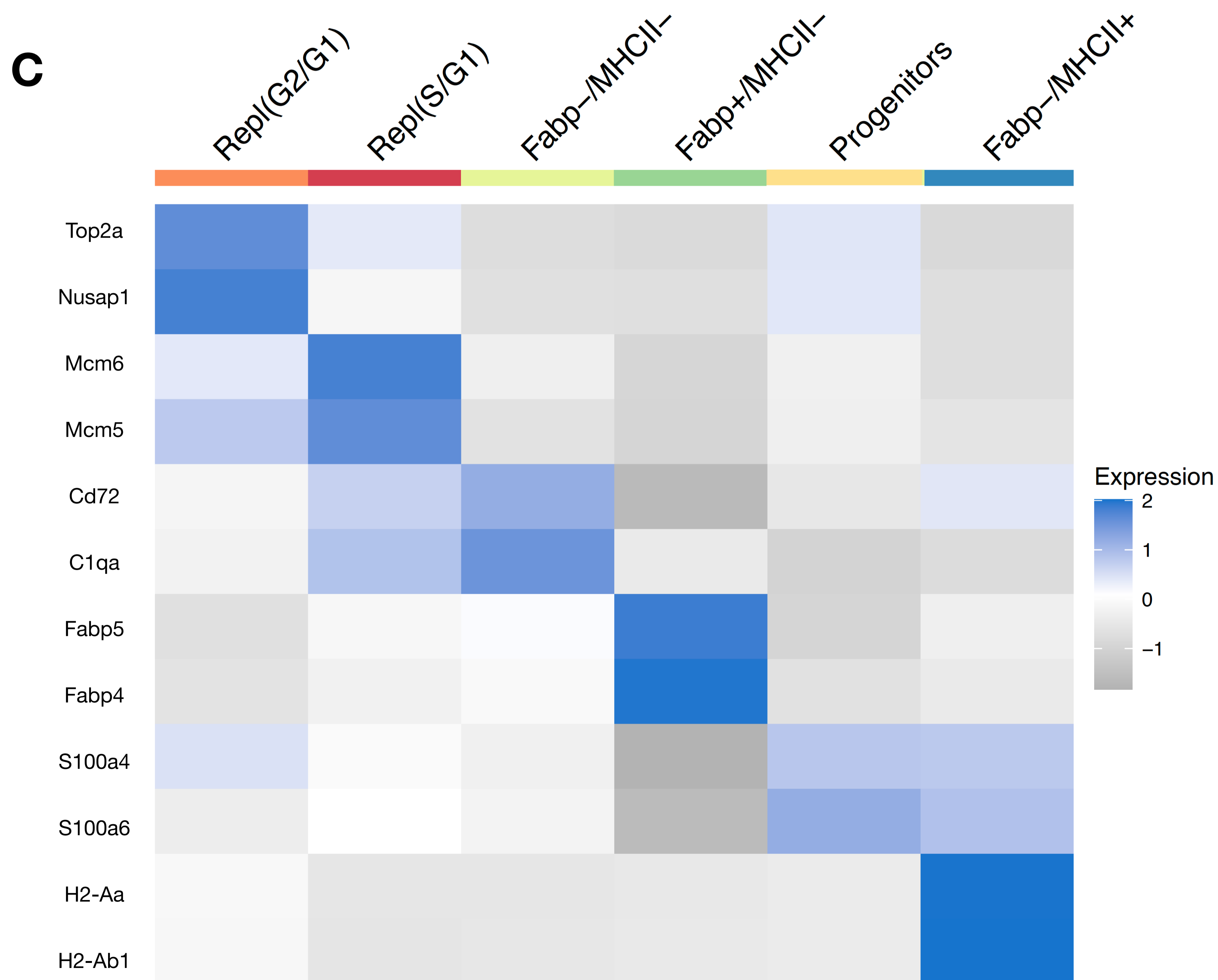

Supplement: Supplementary Figure 1 — Integrated single cell transcriptomes reveal cell-cycling and non-cycling BMDM subsets. (A) Data from all experimental conditions was integrated and clustered. Unsupervised clustering of integrated data (n= 24,377 cells) revealed at least 6 distinct BMDM subsets. Data is dimensionally reduced using UMAP and displayed on a 2D plot to communicate relative similarities in transcriptional profiles between BMDM subsets. (B) Identification of replicating BMDMs from clusters based on cell cycle phase (color-coded legend) using cell cycle sorting. (C) Heatmap of biological replicates averaged, scaled expression of defining 1065 differentially expressed genes for each BMDM subset. Cell cycling subsets (clusters 1-2, red & orange) and non-cycling subsets (clusters 3-6, yellow, green, and blue) are annotated. [file Image_1.pdf]

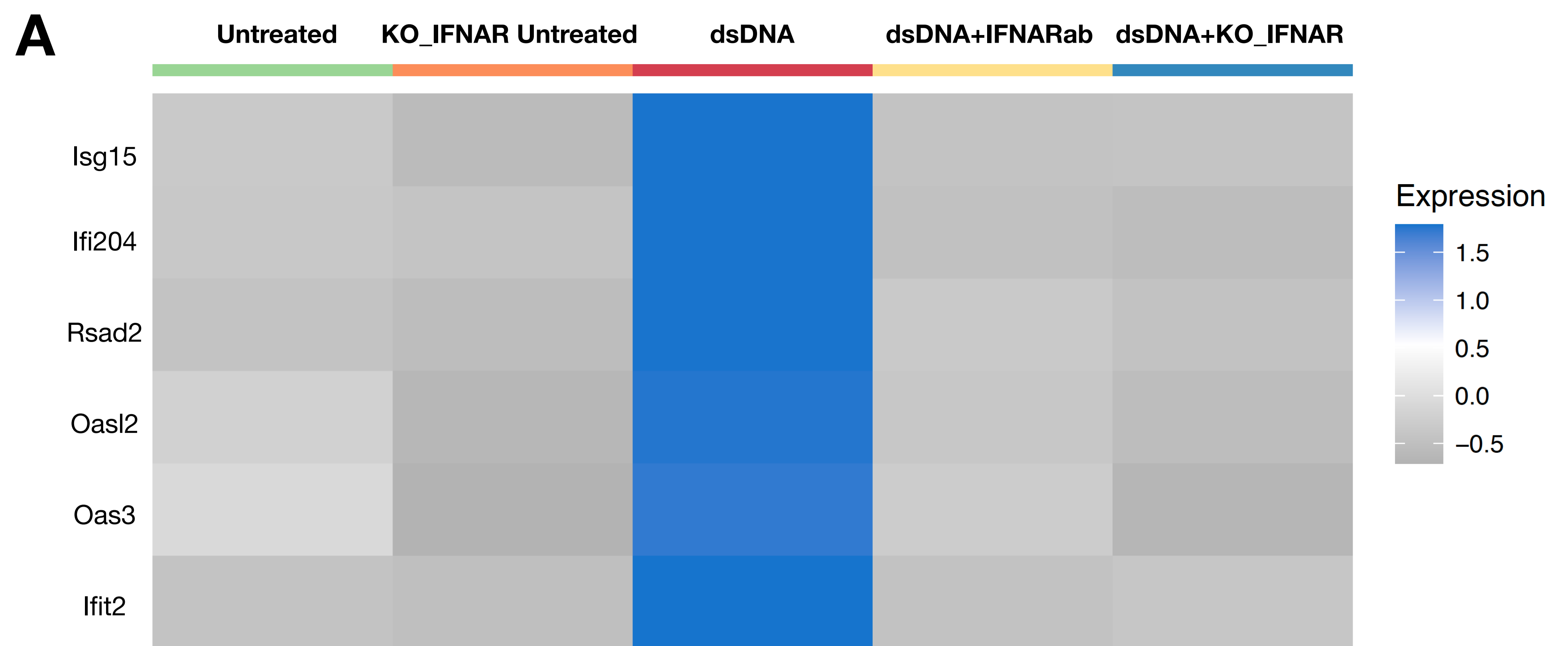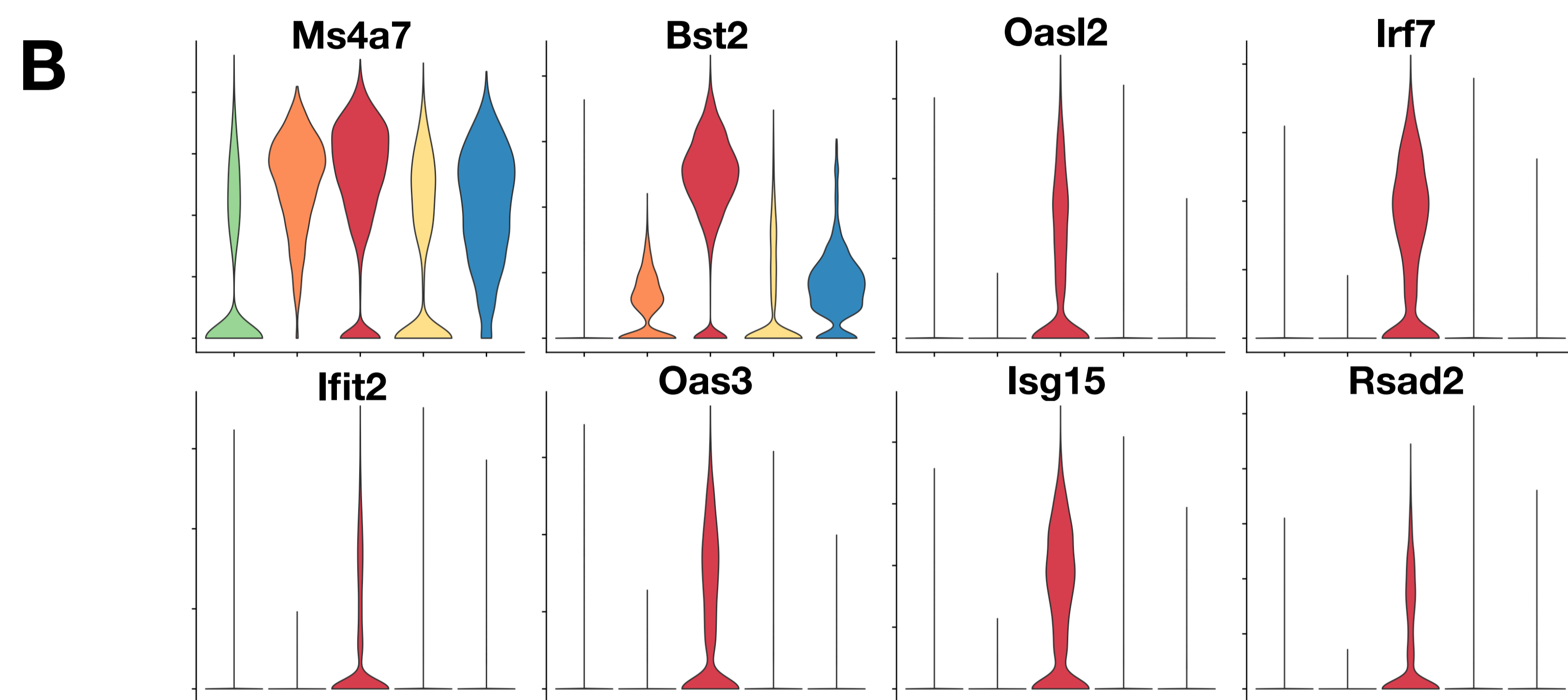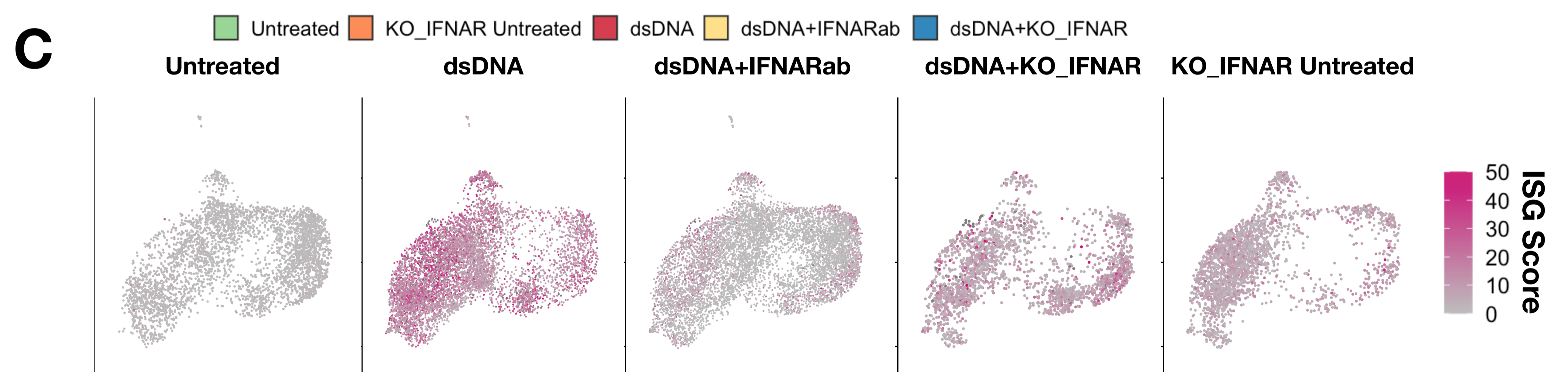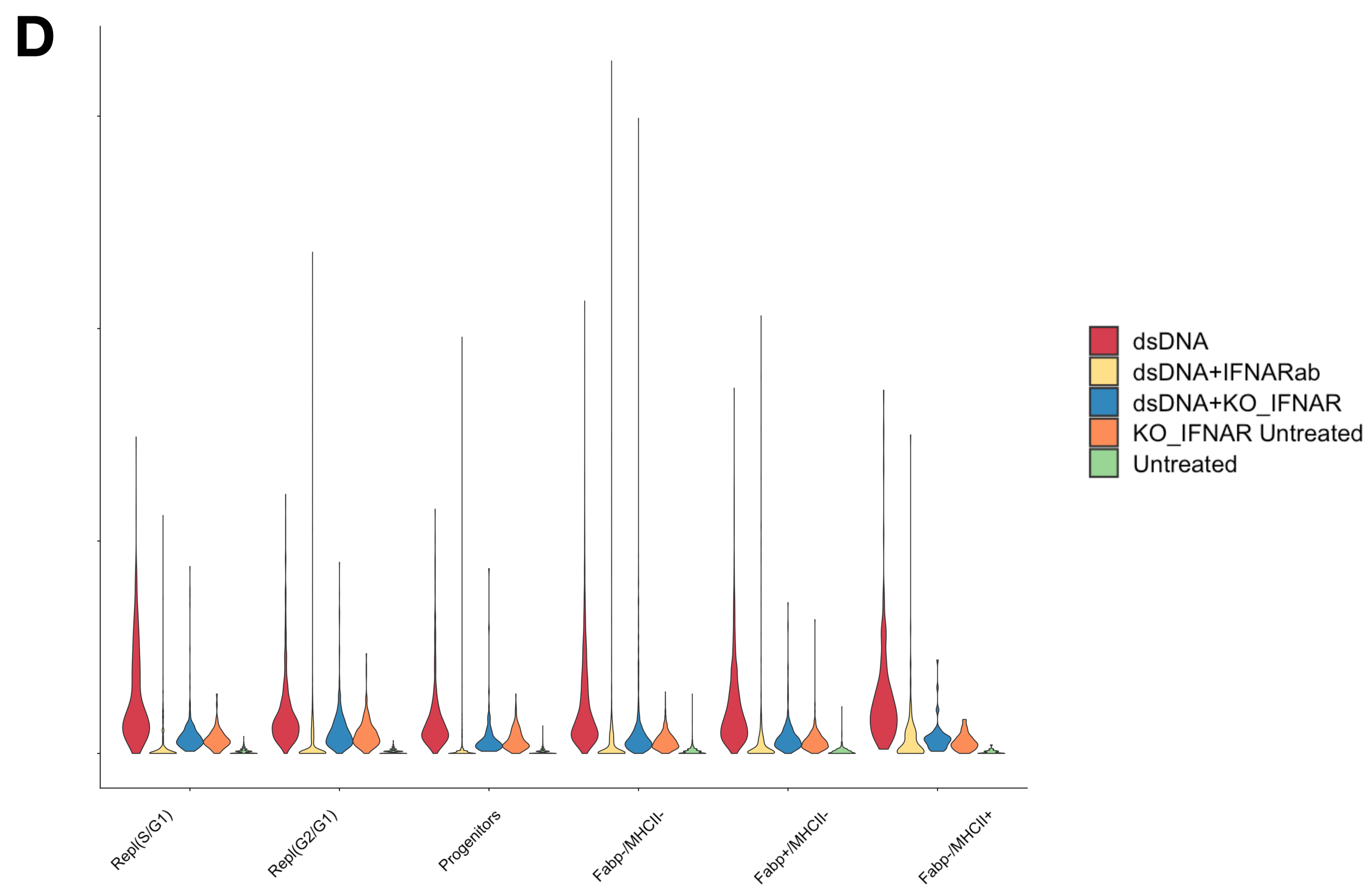

Supplement: Supplementary Figure 2 — Immune stimulatory DNA induces a type I IFN response that can be partially blocked by IFNAR Ab. (A) Heatmap of of biological replicates averaged, scaled expression of ISG genes sorted by experimental condition (1: control, 2: IFNAR KO control, 3: dsDNA, 4: dsDNA+IFNAR Ab, 5: dsDNA+IFNAR KO). (B) Violin plot of Ms4a7, a macrophage marker gene, and several ISGs (Bst2, Oasl2, Irf7, Ifit2, Oas3, Isg15, Rsad2). (C) An ISG Score defined as the summed expression of canonical ISGs was calculated for each cell and displayed as magenta intensity on a UMAP feature plot for each condition. (D) A violin plot of ISG Score was plotted across BMDM subsets and split by experimental condition. [file Image_2.pdf]

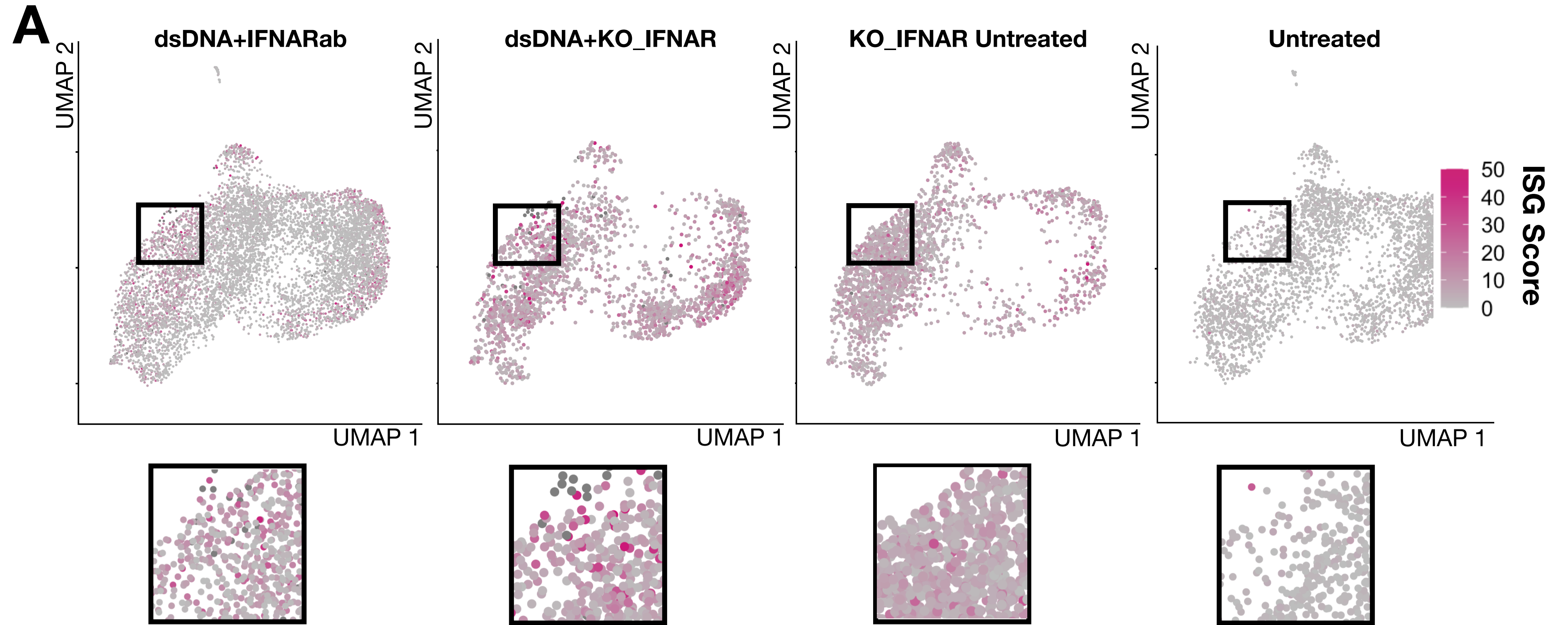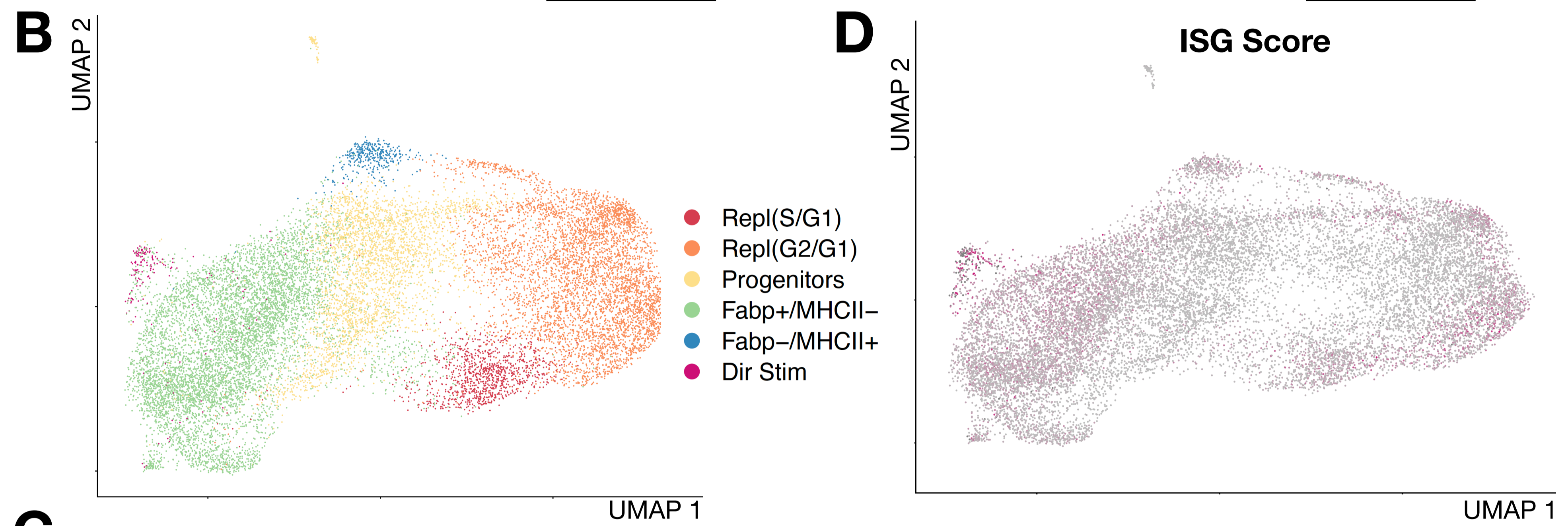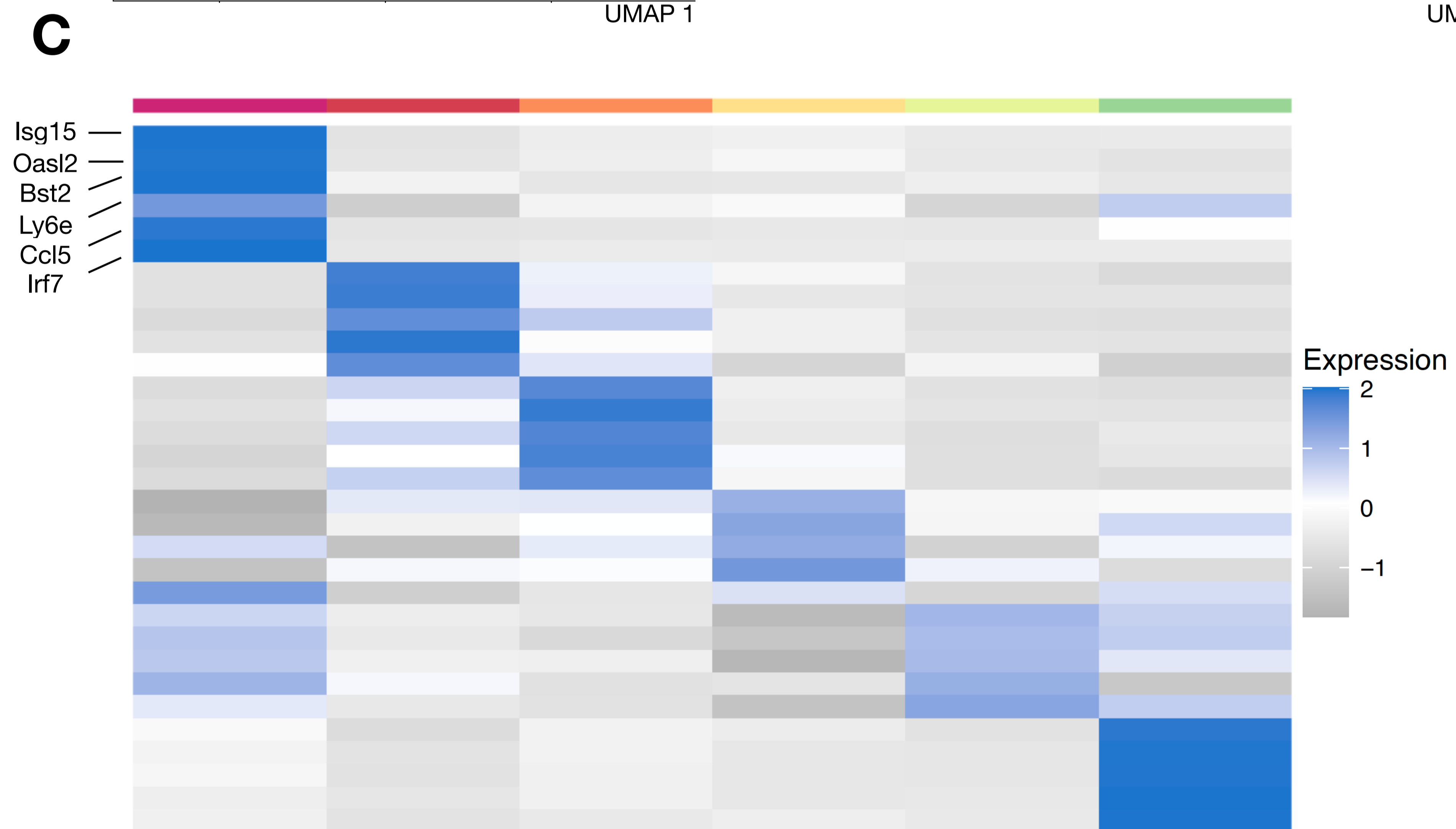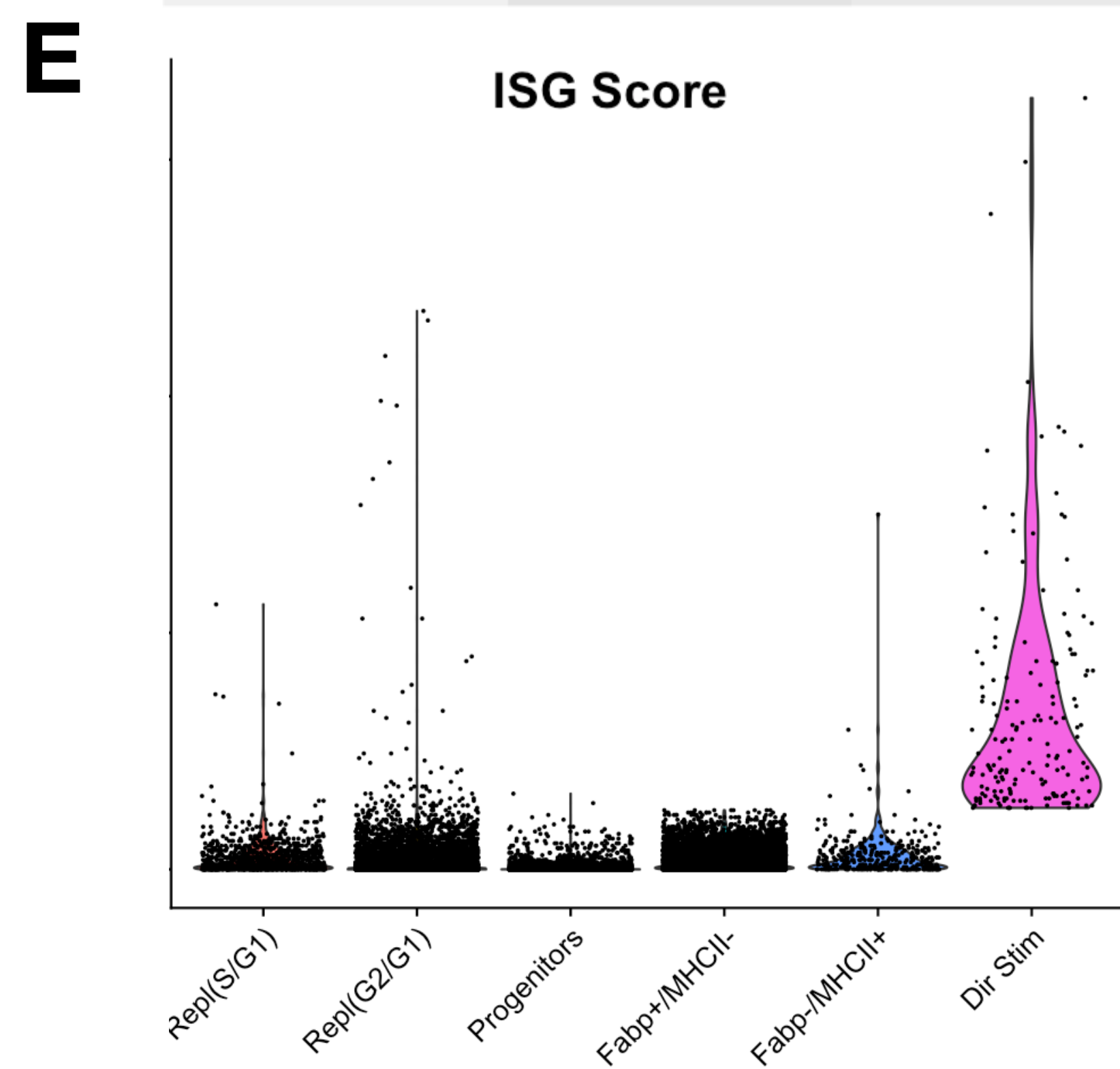

Supplement: Supplementary Figure 3 — IFNAR-independent BMDM responses to immune stimulatory DNA. (A) Feature plot of dsDNA+IFNAR Ab, dsDNA+IFNAR KO, and untreated experimental conditions colored magenta based on ISG Score. (B) Feature plot of integrated and clustered data from dsDNA+IFNAR Ab, dsDNA+IFNAR KO, and Untreated conditions, demonstrating BMDM subsets. (C) Heatmap of biological replicates averaged, scaled expression displaying marker genes for each subset. (D) Feature plot of ISG Score reveals a small cluster expressing ISGs amidst a larger cluster of Fabp4+MHCII- cluster.(E) Violin plot of ISG Score for each BMDM subset from the integration of control and dsDNA+IFNAR Ab conditions. [file Image_3.pdf]

**A**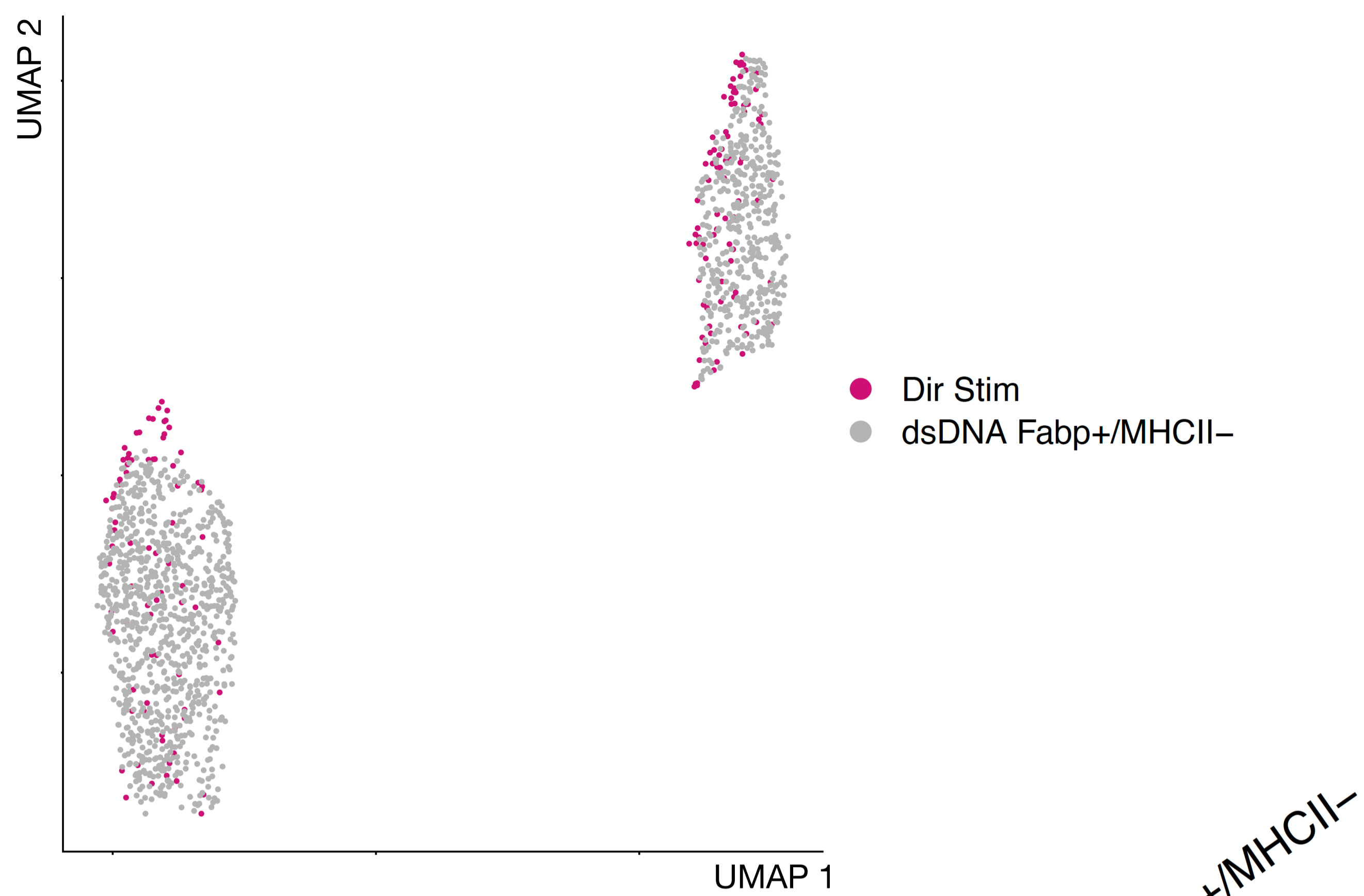**B**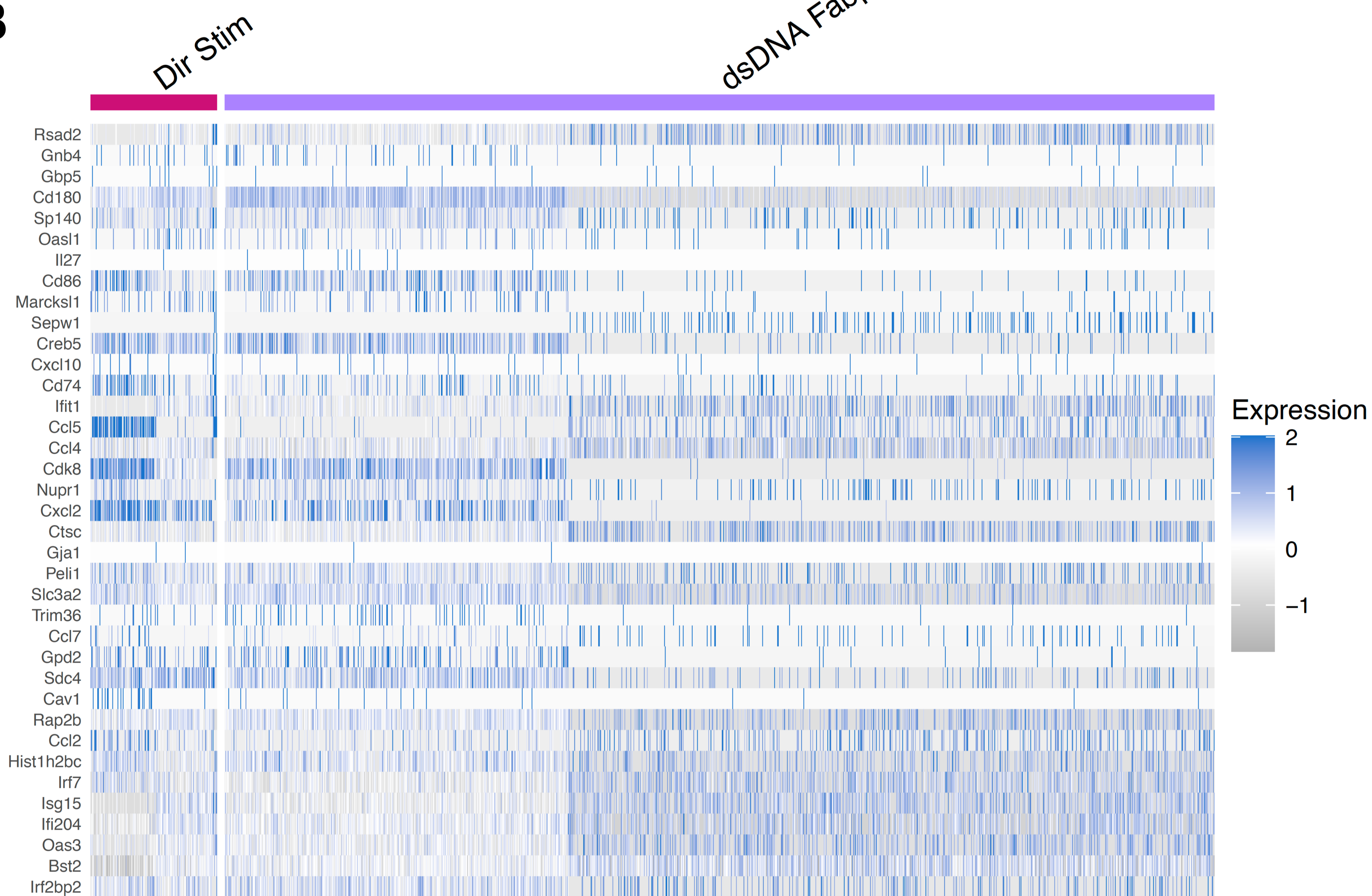**C**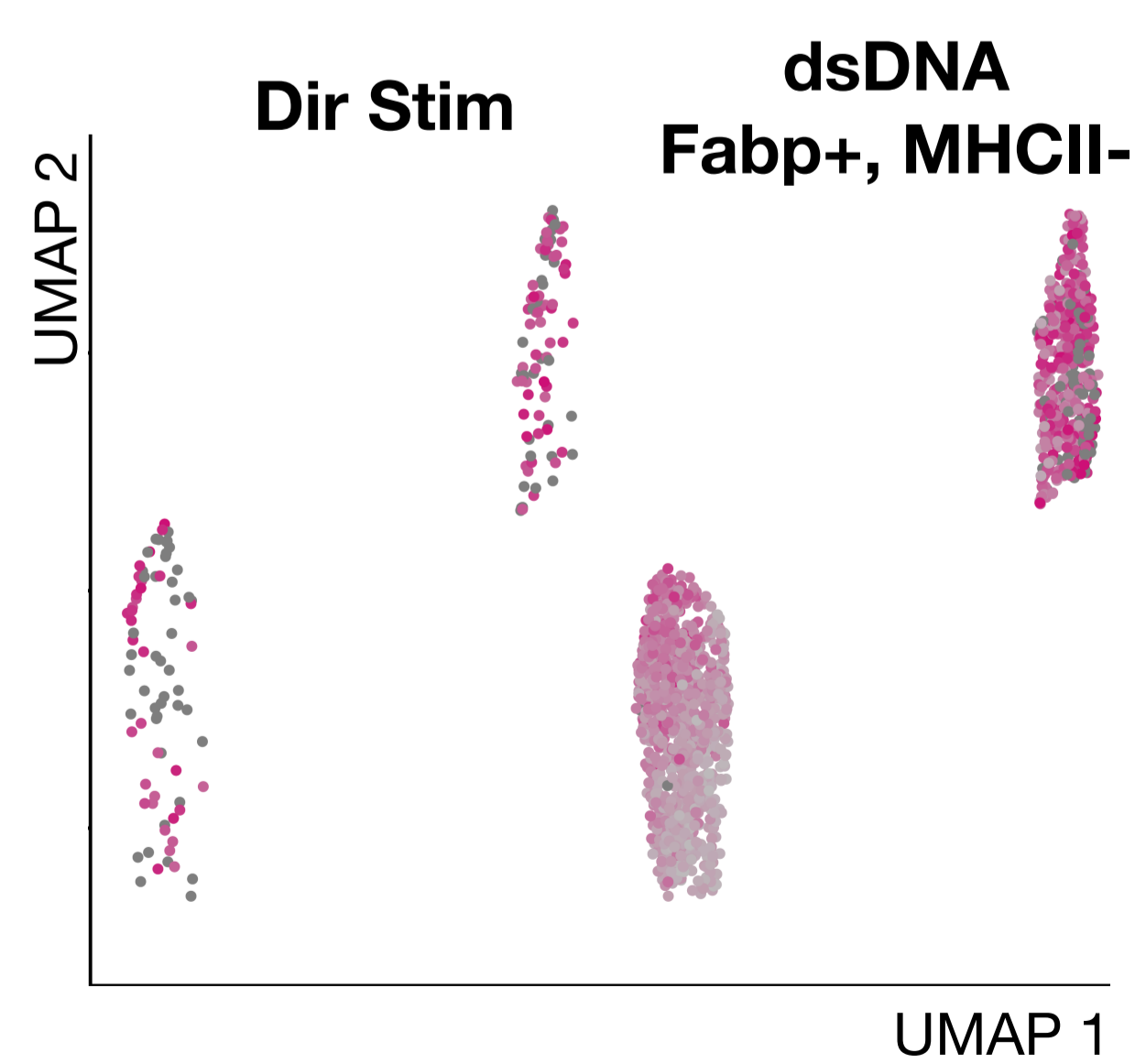**D**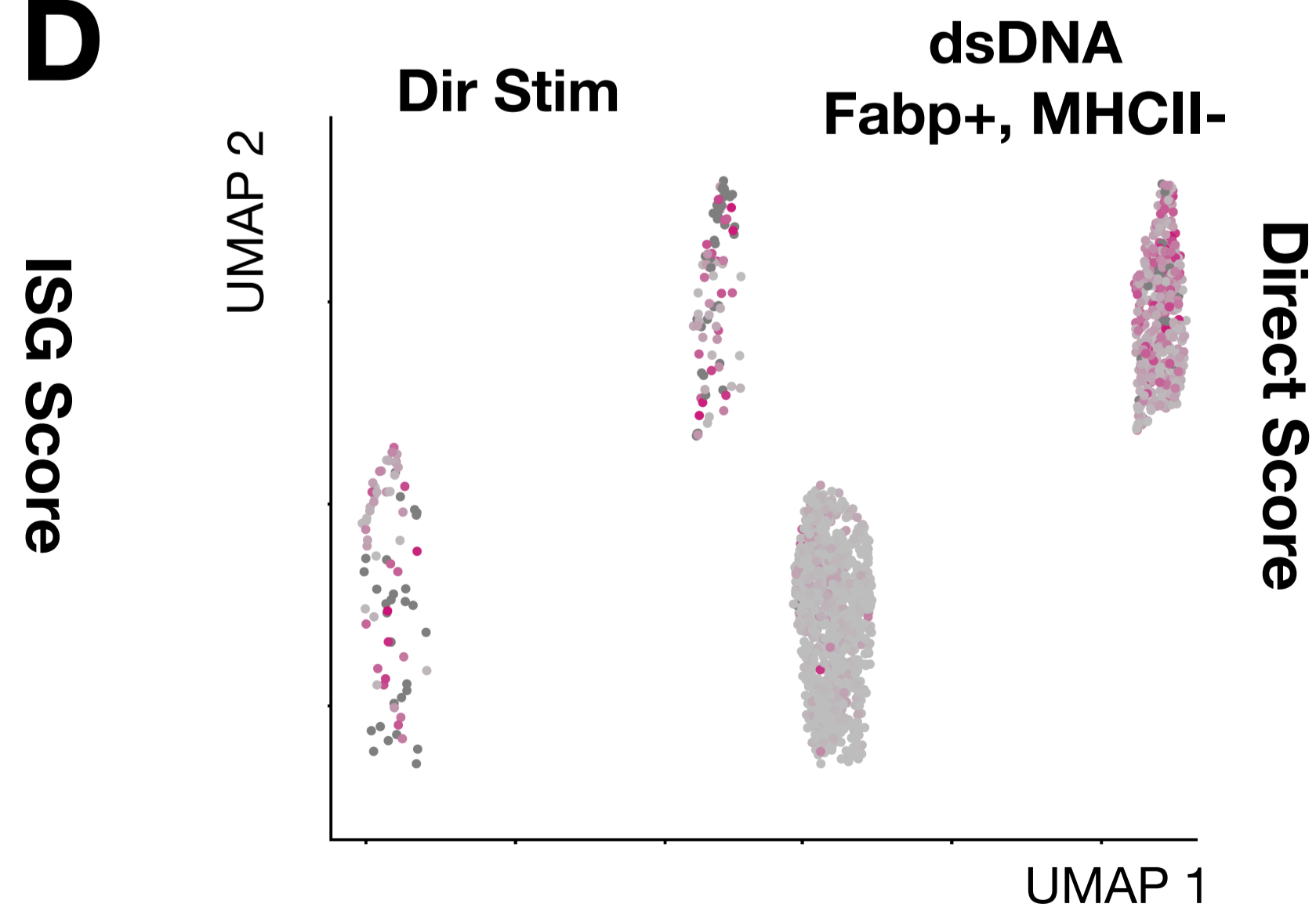

Supplement: Supplementary Figure 4 — Gene expression signature of direct dsDNA-sensing IFNAR-independent BMDMs. (A) Reclustering of Direct Stimulated cells from dsDNA + IFNAR Ab and dsDNA+IFNAR KO conditions integrated with Fabp4+MHCII- BMDMs subset from the dsDNA stimulated experimental condition. (B) Heatmap of Fabp4+MHCII- BMDMs reveals ISG+ and ISG- subsets. The ISG+ subset are the putative direct dsDNA-stimulated macrophages. Differentially expressed genes between ISG+ and ISG- subsets include Ccl5, Cdk8, Cxcl2, and Cd74. C-D) Feature plots comparing (C) ISG Score compared to a (D) DirStim Score (Ccl5, Cdk8, Cxcl2, and Cd74). [file Image_4.pdf]
